# Bias, Study Quality, and Confounding in Temporomandibular Disorder Research Compared to General Orthodontic Studies: A Systematic Review and Meta-Analysis

**DOI:** 10.3390/jcm14248907

**Published:** 2025-12-17

**Authors:** Martin Baxmann, Márton Zsoldos, Krisztina Kárpáti

**Affiliations:** 1 Department of Orthodontics, Faculty of Education and Research, University for Digital Technologies in Medicine and Dentistry, 9516 Wiltz, Luxembourg; 2 Department of Orthodontics and Paediatric Dentistry, Faculty of Dentistry, University of Szeged, 6720 Szeged, Hungarykarpati.krisztina@stoma.szote.u-szeged.hu (K.K.)

**Keywords:** TMD, orthodontic research methodology, risk of bias, confounding in dental research, study quality assessment

## Abstract

**Background/Objectives**: Temporomandibular disorders (TMDs) are a heterogeneous subset of orthodontic conditions with persistent diagnostic and reporting variability. This review compared transparency, reporting quality, and spin prevalence in TMD/TMJ (temporomandibular joint)-focused orthodontic randomized controlled trials (RCTs) versus general orthodontic RCTs. **Methods**: The review followed PRISMA 2020 and was registered in PROSPERO (4201024184). Searches were performed in PubMed/MEDLINE, Embase, CINAHL, ClinicalTrials.gov, and the WHO International Clinical Trials Registry Platform from the earliest available records in each database up to 15 October 2025. Eligible studies were peer-reviewed human orthodontic RCTs. Five transparency indicators (funding disclosure, bias discussion, confounder consideration, protocol registration, reporting-guideline adherence) and five spin indicators (selective focus, unsupported efficacy claims, emphasis on benefits, recommendations despite nonsignificance, “trend toward significance” language) were coded dichotomously. Beta–binomial mixed-effects models compared composite scores between groups, adjusting for publication era, impact factor, and journal clustering. **Results**: Among 874 included trials (840 general, 34 TMD/TMJ-focused), TMD/TMJ-focused studies showed lower adjusted transparency (odds ratio (OR) = 0.58; 95% confidence interval (CI) 0.34–0.99; *p* = 0.047), mainly due to limited registration and incomplete guideline adherence. Predicted transparency proportions were 0.82 for general and 0.73 for TMD/TMJ-focused studies. Composite spin did not differ (OR = 1.05; 95% CI 0.68–1.62; *p* = 0.821), though TMD/TMJ-focused abstracts more often emphasized benefits (OR = 4.62) and recommended interventions despite nonsignificant primary outcomes (OR = 2.83). **Conclusions**: TMD-focused orthodontic trials exhibited lower transparency and a distinct pattern of interpretive spin, particularly a greater tendency to emphasize benefits or recommend interventions despite non-significant results, compared with general orthodontic research.

## 1. Introduction

TMDs encompass a heterogeneous group of conditions affecting the TMJ, masticatory muscles, and associated craniofacial structures [1,2]. These disorders are commonly characterized by pain, joint dysfunction, limitations in mandibular movement, and occlusal discrepancies, impacting the quality of life for a substantial proportion of the population [3,4,5]. The relationship between TMD and orthodontic treatment has long been a subject of debate, with conflicting evidence regarding whether orthodontic interventions influence the onset or severity of TMD symptoms [6,7,8,9]. While some studies suggest a potential association between orthodontic procedures and the development or exacerbation of TMD, others report no causal link, highlighting the need for rigorous, unbiased research to inform clinical decision-making.

The scientific literature addressing TMD within orthodontics has grown considerably in recent decades, reflecting both the clinical importance and persistent controversy surrounding this topic [10,11]. This body of work spans research on TMD etiology, diagnostic frameworks, management protocols, and treatment outcomes [2,3,12]. Concurrently, multiple appraisals have highlighted marked methodological heterogeneity and reporting limitations in TMD trials, including small sample sizes, inconsistent diagnostic criteria, and incomplete harm reporting [2,13,14]. Such variability, together with differences in patient selection and frequent reliance on patient-reported outcomes, hinders evidence synthesis and reproducibility [15].

Unlike general orthodontic research, which primarily focuses on outcomes related to skeletal and dental alignment, occlusal correction, and treatment efficiency, TMD studies often integrate neuromuscular, psychosocial, and behavioral dimensions [16,17]. The multifactorial nature of TMD pathophysiology, combined with inconsistent inclusion criteria and heterogeneous study designs, increases the risk of confounding and interpretive bias [17,18,19]. These complexities hinder the standardization of study protocols and the synthesis of findings, ultimately affecting the reliability and validity of conclusions drawn from the TMD literature.

In recent years, increasing attention has been paid to research integrity in orthodontics, including transparency in reporting, the disclosure of funding sources, protocol registration, and adherence to recognized reporting guidelines [20,21,22]. Interpretative practices such as ‘spin,’ defined as selectively presenting, emphasizing, or framing results to make findings appear more favorable, have been documented as a form of reporting bias that can distort the evidence base and mislead clinicians [23,24,25]. These concerns are particularly relevant in the context of TMD research, where study heterogeneity and subjective outcomes are common.

This issue is particularly relevant because clinicians frequently rely on abstracts as their primary source of information. Multiple studies have shown that physicians read only the abstract for roughly two-thirds of the articles they encounter, using it to determine clinical relevance or whether to pursue the full text [26,27,28,29]. Time constraints, information overload, and limited access contribute to this selective reading behavior, and when full articles are read, attention often centers on the conclusion or results rather than methodological details [28,30]. Because abstracts may omit important limitations or inconsistencies, overinterpretation or “spin” in abstracts has a disproportionate potential to shape clinical impressions and decision-making [26,27,29].

Nonetheless, existing meta-research in orthodontics has typically examined transparency and spin either within single subspecialties or across heterogeneous collections of randomized trials, without explicitly contrasting TMD/TMJ-focused orthodontic RCTs with general orthodontic RCTs [20,21,22,23,24,25]. As a result, it remains unclear whether TMD/TMJ trials are characterized by systematically different transparency profiles or spin patterns compared with the broader orthodontic literature. Clarifying this gap is important for interpreting the strength of evidence in a clinically controversial area.

Accordingly, the present systematic review and meta-analysis aimed to assess and compare the methodological quality, transparency, reporting practices, and prevalence of spin in studies focused on TMD versus general orthodontic research. By evaluating patterns of study rigor and interpretive reporting across these two domains, this review sought to determine whether systematic methodological differences exist and to clarify whether the evidence base for TMD in orthodontics adheres to the principles of transparency and evidence-based practice.

## 2. Materials and Methods

### 2.1. Protocol and Reporting Framework

This systematic review and meta-analysis followed the PRISMA 2020 guidelines (Supplementary checklist) to ensure methodological transparency and reproducibility at each stage. The protocol was prospectively registered in the International Prospective Register of Systematic Reviews (PROSPERO 4201024184). After registration and before data extraction, two minor protocol deviations were introduced. First, the TMD/TMJ search strategy was broadened to include additional synonyms and free-text variants for temporomandibular terminology to improve the retrieval of orthodontic TMD trials. Second, the author list was updated (one initially listed author withdrew and another joined the review team) to reflect the final study team. These modifications did not alter the review question, eligibility criteria, outcomes, or planned analytical framework. A summary of protocol amendments, with dates and justifications, is provided in Supplementary Table S4.

### 2.2. Eligibility Criteria

Eligible studies were limited to human clinical research in orthodontics published in peer-reviewed journals. Randomized controlled trials (parallel, split-mouth, crossover, and cluster designs), quasi-randomized controlled trials, and prospective controlled clinical trials were eligible. Quasi-randomized designs were defined as trials using a systematic but non-random allocation method (e.g., alternation, date of birth, or clinic day). Trials in which allocation clearly reflected clinician or patient preference without a predefined allocation rule were considered non-randomized and were excluded.

Studies were categorized as either TMD/TMJ-focused or general orthodontic based on the primary research question, intervention type, and outcomes reported. Trials were classified as TMD/TMJ-focused when the main objective was prevention or management of TMD/TMJ symptoms or dysfunction using orthodontic, orthopedic, or occlusal interventions. General orthodontic studies encompassed trials evaluating malocclusion correction, tooth movement, growth modification, anchorage reinforcement, or treatment efficiency, excluding airway-related research already examined elsewhere. When authors explicitly specified primary versus secondary outcomes, inclusion and classification decisions were based on the stated primary outcome. In the absence of an explicit designation, the primary outcome was inferred from the outcome emphasized in the study objectives and abstract conclusions. All included studies can be viewed in Supplementary Table S5.

Exclusion criteria comprised non-randomized observational studies, retrospective designs, secondary analyses, case series, animal or in vitro studies, narrative reviews, editorials, commentaries, book chapters, and studies lacking full-text availability. Trials were excluded as “solely non-orthodontic” when all primary outcomes related exclusively to pain modulation, oral hygiene, remineralization, or other non-orthodontic domains, even if orthodontic appliances were used. Studies in which such outcomes were secondary, but at least one primary orthodontic or occlusal outcome was evaluated, were eligible.

### 2.3. Information Sources and Search Strategy

A comprehensive search was designed and executed in consultation with a health sciences librarian. Electronic databases searched from the earliest available records in each database up to 15 October 2025 included PubMed/MEDLINE, ICTRP, EMBASE, CT.gov and CINAHL. The search was structured around two complementary frameworks: one identifying general orthodontic trials and one identifying TMD/TMJ orthodontic trials. Search strings combined controlled vocabulary (e.g., MeSH) and free-text keywords using Boolean operators AND/OR. No language, date, or geographic restrictions were applied. The PubMed search strategy for general orthodontics, provided in full below, served as the exemplar; database-specific headings and syntax were adapted for the remaining sources: ((orthodontic[Title/Abstract] OR orthodontics[MeSH Terms] OR “orthodontic appliance”[Title/Abstract] OR “orthodontic appliances”[Title/Abstract] OR “interceptive orthodontics”[Title/Abstract] OR “clinical orthodontics”[Title/Abstract] OR “dental braces”[Title/Abstract] OR “dental bracket”[Title/Abstract])) AND (airway[Title/Abstract] OR “sleep apnea”[Title/Abstract] OR “upper airway”[Title/Abstract] OR OSA[Title/Abstract] OR snoring[Title/Abstract] OR breathing[Title/Abstract] OR respiratory[Title/Abstract] OR “pharyngeal airway”[Title/Abstract]) AND (randomized controlled trial[Publication Type] OR controlled clinical trial[Publication Type] OR randomized[Title/Abstract] OR randomized[Title/Abstract] OR trial[Title/Abstract] OR “clinical trial”[Title/Abstract] OR split-mouth[Title/Abstract] OR prospective[Title/Abstract] OR “quasi-randomized”[Title/Abstract] OR “quasi randomized”[Title/Abstract]) NOT (animals[MeSH Terms] NOT humans[MeSH Terms])

The PubMed search strategy for TMJ/TMD-focused orthodontic trials, reported in full below, served as the template strategy. For Embase, CINAHL, ClinicalTrials.gov, and ICTRP, the same conceptual structure and keywords were translated into database-specific controlled vocabulary (e.g., Emtree, CINAHL Headings) and syntax (field tags, truncation, and Boolean operators). The complete PubMed strategies and this description allow replication of the search approach; full database-specific strings are available in Supplementary File S1.

The PubMed search strategy was as follows: (“Temporomandibular Joint Disorders”[Mesh] OR “Temporomandibular Joint”[Mesh] OR “Craniomandibular Disorders”[Mesh] OR temporomandibular[tiab] OR TMJ[tiab] OR TMD[tiab] OR craniomandibular[tiab]) AND (“Orthodontics”[Mesh] OR “Malocclusion”[Mesh] OR “Dental Occlusion”[Mesh] OR orthodontic[tiab] OR orthodontics[tiab] OR malocclusion[tiab] OR dentofacial[tiab] OR occlusion[tiab]) AND (therapy[tiab] OR treatment[tiab] OR intervention[tiab] OR appliance[tiab] OR splint[tiab] OR expansion[tiab] OR functional[tiab] OR mandibular[tiab] OR maxillary[tiab]) NOT (“Systematic Review”[Publication Type] OR “Meta-Analysis”[Publication Type] OR review[pt] OR editorial[pt] OR comment[pt] OR letter[pt] OR case reports[pt] OR protocol[tiab] OR arthroplasty[tiab] OR prosthesis[tiab] OR replacement[tiab] OR animals[mh] NOT humans[mh])

### 2.4. Study Selection

All search results were imported into Covidence for deduplication and screening. Two reviewers independently screened titles and abstracts. Potentially eligible records proceeded to full-text review. Disagreements were resolved by discussion or third-reviewer adjudication. Inter-reviewer agreement was quantified using Cohen’s κ, which indicated substantial agreement for title/abstract screening (κ = 0.76) and near-perfect agreement for full-text eligibility (κ = 0.87).

**Figure 1:**
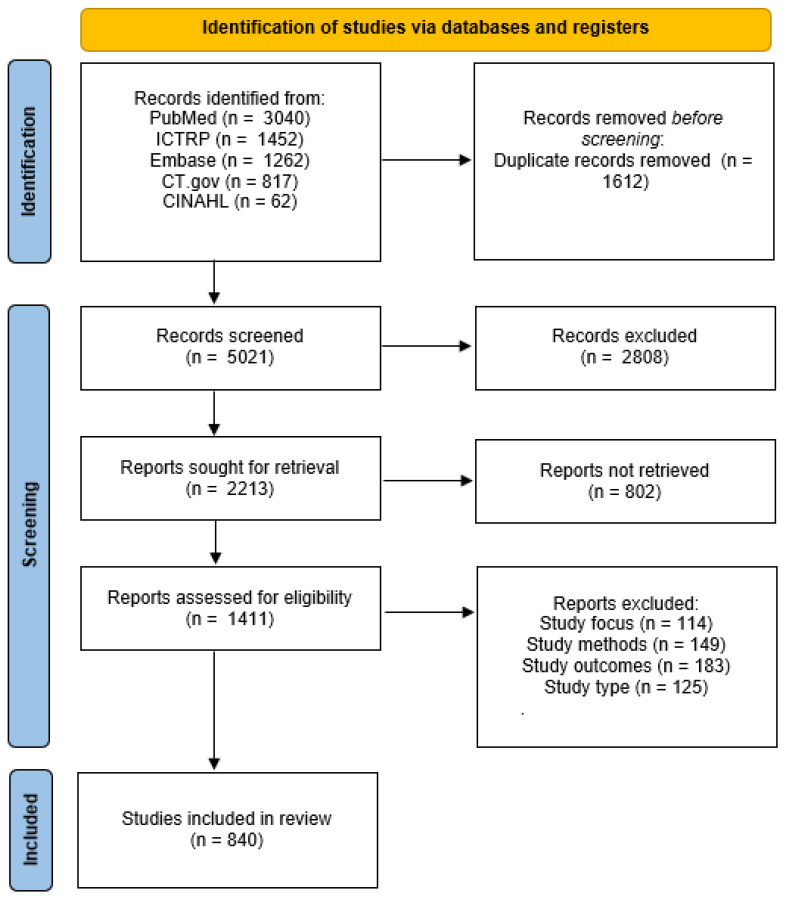
PRISMA flow diagram of general orthodontic studies included in the review.

**Figure 2:**
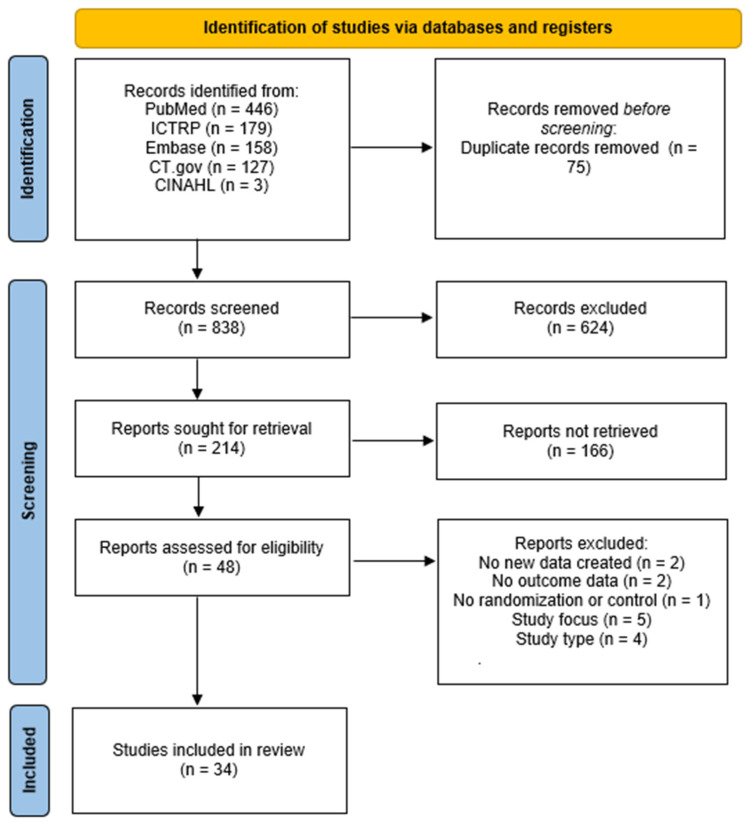
PRISMA flow diagram of TMD/TMJ-focused orthodontic studies included in the review.

Reasons for full-text exclusions were recorded and summarized in the PRISMA flow diagrams for TMD/TMJ and general orthodontic literature (Figure 1 and Figure 2). The identification stage in each diagram specifies the number of records retrieved from each database (PubMed/MEDLINE, Embase, CINAHL, ClinicalTrials.gov, and ICTRP). The complete list of full-text exclusions can be found in Supplementary Tables S1 and S2.

### 2.5. Data Extraction and Variables

Data extraction was performed independently and in duplicate using a piloted, codebook-based Excel template. Extracted variables included authors, year of publication, journal, impact factor, study design, and variables describing reporting practices. Transparency was defined as the completeness and openness with which key methodological and reporting practices were described in the trial reports. Specifically, we assessed five indicators: (1) funding disclosure; (2) discussion of potential bias; (3) consideration of confounders; (4) registration of a study protocol or trial; and (5) adherence to recognized reporting guidelines (e.g., CONSORT, PRISMA). Funding disclosure was coded “yes” when any statement about study funding or the absence of funding was provided. Discussion of bias was coded “yes” when the manuscript explicitly addressed risk of bias, internal validity, or methodological limitations. Confounder consideration was coded “yes” when potential confounding variables were prespecified, incorporated into the analysis (e.g., adjusted models), or discussed as possible sources of distortion. Protocol registration was coded “yes” when a registry or protocol identifier (e.g., ClinicalTrials.gov, PROSPERO) was reported. Reporting-guideline adherence was coded “yes” when authors explicitly stated following a named reporting guideline (e.g., CONSORT, PRISMA or related EQUATOR-listed tools).

Spin was conceptualized as an interpretive distortion in how results were framed in the abstract, distinct from whether core methods were transparently reported in the full text. Spin was defined as the presence within the abstract of any of five ways in which the findings were presented more favorably than justified by the primary outcomes, adapted from established spin frameworks in dentistry and orthodontics [23,24,25]: (1) selective focus on statistically significant findings (primary non-significant results downplayed or omitted while significant secondary or subgroup findings are emphasized); (2) claims of efficacy despite non-significant results (stating that an intervention is effective or superior despite non-significant primary outcomes); (3) emphasis on secondary benefits despite nonsignificance (explicit acknowledgement of non-significant primary outcomes but focusing the abstract on favorable secondary trends or outcomes); (4) recommendation of treatment despite non-significant results (concluding that an intervention should be adopted or recommended although primary outcomes are non-significant); and (5) “trend toward significance” language (phrases such as “borderline significant”, “approached significance”, or “trend toward significance” used to imply meaningful benefit).

Each item was coded dichotomously (1 = yes, 0 = no) according to these predefined decision rules. Composite scores (0–5) were computed separately for transparency and spin. Transparency variables were coded from full texts, and spin variables were coded from abstracts only. All disagreements were resolved by consensus after independent coding. The full dataset, including all study-level variables and coding decisions, is provided in Supplementary Table S3.

### 2.6. Data Analysis

Composite transparency and spin scores were analyzed as counts out of five using beta–binomial mixed-effects models with a logit link. Fixed effects included publication-era group (ordered categorical) and log(1 + impact factor) modeled as a natural spline (3 degrees of freedom), chosen a priori to allow moderate non-linearity while avoiding overfitting; journal was modeled as a random intercept. Model convergence was assessed by inspection of optimization and gradient diagnostics, and random-effects variance components were checked for boundary estimates suggesting overfitting. Adjusted odds ratios (ORs) with 95% confidence intervals (CIs) were reported alongside marginal predicted probabilities and risk differences.

The Holm procedure controlled familywise error across the two primary outcomes (total transparency and total spin); Holm was selected as a step-down alternative to Bonferroni that maintains strong familywise error control while being less conservative for a small number of correlated endpoints. Each item-level indicator was examined using mixed-effects logistic regression with the same covariates; bias-reduced (Firth) logistic regression was applied for quasi-separation. The Benjamini–Hochberg correction controlled false-discovery rates within each domain, balancing type I error control with adequate power for multiple item-level tests.

Overdispersion, model fit, and intraclass correlation coefficients (ICCs) were evaluated for each model to assess residual heterogeneity and the magnitude of journal-level clustering. Sensitivity analyses included proportional-odds models, linear versus spline forms of impact factor, and models with cluster-robust standard errors based on sandwich-type variance estimators with clustering by journal. Exploratory analyses assessed correlations between impact factor and total transparency or spin using Kendall’s τ-b (cluster-bootstrap CIs) and partial rank correlations adjusted for group and publication era. Analyses were conducted in R (version 4.4.2) using the glmmTMB, ordinal, emmeans, logistf, ggplot2, and related packages, with two-sided α = 0.05.

### 2.7. Risk of Bias and Reporting-Bias Assessment

Because this review examines reporting practices rather than treatment effects, traditional clinical risk-of-bias tools (e.g., RoB 2, ROBINS-I) were not applicable. These instruments assess internal validity of clinical effect estimates—such as randomization, blinding, and outcome-measurement methods—which are not outcomes of interest in a meta-research study focused on transparency and interpretive spin. Instead, risk of bias was operationalized at the reporting level through the transparency indicators described in Section 2.5.

Classification bias was addressed through predefined coding rules, piloting of the extraction form, and independent duplicate screening and data extraction with third-reviewer adjudication. Potential reporting bias related to missing or unavailable preregistration information was considered descriptively by recording whether studies provided an accessible protocol or registry entry during data extraction. Because no synthesis of effect sizes was conducted, conventional quantitative publication-bias methods (e.g., funnel-plot-based or regression-based approaches) were not applicable to the outcomes assessed in this review.

## 3. Results

A total of 6633 general orthodontic records were identified through database searching for general orthodontic literature, of which 1612 duplicates were removed before screening. After screening 5021 unique titles and abstracts, 2808 were excluded for irrelevance. The full text of 2213 reports was sought, with 802 unobtainable. Of the 1411 reports assessed for eligibility, 571 were excluded due to an ineligible study focus (n = 114), methods (n = 149), outcomes (n = 183), or study type (n = 125). Ultimately, 840 general orthodontic randomized or controlled trials met the inclusion criteria and were analyzed (Figure 1).

For the TMD/TMJ-focused subset, 913 records were identified, and 75 duplicates were removed before screening. Of the remaining 838 records screened, 624 were excluded, and 166 reports were not retrieved. Forty-eight full-text reports were evaluated, and 14 were excluded because they presented no new data (n = 2), lacked outcome data (n = 2), had no randomization or control (n = 1), were outside the review focus (n = 5), or did not meet study type requirements (n = 4). Thirty-four TMD/TMJ clinical trials satisfied all inclusion criteria (Figure 2).

Across the 874 included studies, mean total transparency scores were 0.66 for general orthodontic and 0.62 for TMD/TMJ trials. Beta–binomial mixed-effects modeling, adjusted for publication era, journal impact factor, and clustering by journal, demonstrated significantly lower transparency among TMD/TMJ studies (OR = 0.58; 95% CI 0.34–0.99; *p* = 0.047). Adjusted marginal predictions indicated transparency proportions of 0.82 for general and 0.73 for TMD/TMJ studies. Item-level comparisons (Table 1) showed that both groups most frequently considered confounders (general 83.6%, TMD/TMJ 73.5%) and discussed potential risk of bias (69.0% and 79.4%, respectively). By comparison, protocol registration and formal reporting-guideline adherence were the main areas of deficiency: only 55.5% of general orthodontic trials and 26.5% of TMD/TMJ trials reported a registered protocol, and 71.4% and 58.8% of trials, respectively, explicitly stated use of a reporting guideline.

Item-level comparisons (Table 1) showed that TMD/TMJ trials were markedly less likely to register a study protocol (26.5% vs. 55.5%; OR = 0.15; 95% CI 0.07–0.30; *p* < 0.001) or follow formal reporting guidelines (58.8% vs. 71.4%; OR = 0.38; 95% CI 0.21–0.69; *p* = 0.001). Differences in funding disclosure, risk-of-bias discussion, and confounder consideration were not statistically significant.

Composite spin scores did not differ between groups (OR = 1.05; 95% CI 0.68–1.62; *p* = 0.821), with adjusted predicted spin proportions of 0.31 for general and 0.32 for TMD/TMJ studies (Table 2). Nonetheless, distinct spin patterns emerged at the item level (Table 1). General orthodontic abstracts more frequently focused selectively on statistically significant findings (Spin 1: 65.5% vs. 0.00% in TMD/TMJ trials), whereas TMD/TMJ abstracts more often acknowledged non-significant primary outcomes while emphasizing secondary benefits (Spin 3: 37.0% of general vs. 73.5% of TMD/TMJ trials) and recommending the intervention despite non-significant results (Spin 4: 31.0% vs. 55.9%). Unsupported efficacy claims despite non-significant results (Spin 2) and “trend toward significance” language (Spin 5) were relatively uncommon in both groups (<10% in each group). Later publication eras and higher journal impact factors were positively associated with transparency but not with spin outcomes (Table 2).

## 4. Discussion

This review compared reporting transparency and interpretive spin between TMD/TMJ-focused orthodontic trials and general orthodontic RCTs. While overall transparency was moderate across both domains, TMD/TMJ studies demonstrated significantly lower adjusted transparency scores, driven primarily by under-reporting of protocol registration and incomplete adherence to reporting guidelines. In comparison, composite spin prevalence was similar between groups, but the nature of spin differed. Abstracts of TMD/TMJ trials more frequently acknowledged non-significant primary outcomes while emphasizing secondary benefits or recommending the intervention, whereas general orthodontic abstracts more often focused selectively on statistically significant findings. These patterns suggest that although orthodontic research overall has improved in methodological integrity, TMD/TMJ studies may remain particularly vulnerable to interpretive framing that obscures uncertainty.

The transparency profile observed in this review mirrors earlier meta-research in orthodontics and related dental fields, where fewer than two-thirds of RCTs disclose funding sources, register protocols, or fully adhere to CONSORT reporting standards [20,21]. Lower transparency in TMD/TMJ trials likely reflects diagnostic and methodological variability inherent to this field, where multifactorial etiologies, small sample sizes, and subjective outcome measures hinder reproducibility and preregistration. Incomplete registration and inconsistent adherence to reporting guidelines may therefore perpetuate uncertainty surrounding orthodontic involvement in TMD by impeding the accumulation of high-quality, comparable evidence. The persistence of these challenges underscores structural differences between TMD/TMJ and general orthodontic research environments.

The pattern of spin identified here aligns with findings from prior analyses of dental and orthodontic literature, which reported frequent selective emphasis and positive framing despite non-significant results [24,25]. Such practices are concerning because, as outlined in the Introduction, clinicians frequently base initial impressions on abstracts rather than full texts. Importantly, the association between later publication era and improved transparency observed here mirrors the upward trend reported in recent orthodontic meta-research following widespread adoption of PRISMA, CONSORT, and EQUATOR guidelines. Studies published in higher-impact journals also demonstrated greater adherence to transparency indicators, a trend consistent with other biomedical fields. Despite these advances, the continued absence of preregistration and persistent interpretive bias indicate that full alignment with open-science and reproducibility standards remains incomplete.

### 4.1. Limitations

Several limitations should be considered when interpreting these findings. First, the analysis included only peer-reviewed literature indexed in major databases and trial registries. A formal evaluation of publication or availability bias (for example, selective non-publication of registered trials or discrepancies between registered and published outcomes) was not performed, so such biases cannot be ruled out. Second, although data extraction was performed with predefined coding rules, binary scoring of transparency and spin variables cannot capture the full nuance of reporting quality or abstract framing. Third, because the review examined reporting practices rather than treatment effects, conventional domain-based risk-of-bias tools were not applied, limiting comparability with clinical meta-analyses.

The number of TMD/TMJ trials was considerably smaller than that of general orthodontic studies (34 vs. 840), which reduces power to detect modest group differences, increases uncertainty around estimates for the TMD/TMJ group, and necessitates treating “TMD/TMJ vs. general” as a dichotomous grouping factor rather than modeling more granular TMD subtypes. As a result, estimates for the TMD/TMJ-focused subset should be interpreted with caution. Finally, although models accounted for journal clustering and publication era and underwent basic diagnostic checks, residual confounding from unmeasured study characteristics, such as funding magnitude, institutional environment, or author experience, cannot be excluded.

### 4.2. Implications for Practice and Future Directions

These results carry implications for researchers, clinicians, and journal editors. For investigators, the consistent underreporting of protocol registration and guideline adherence emphasizes the need for greater engagement with open-science practices. Trial registration, protocol transparency, and standardized reporting frameworks remain essential for improving reproducibility and reducing bias. For editors and peer reviewers, consistent enforcement of CONSORT-aligned abstract structures and mandatory registration policies may further mitigate interpretive spin.

Clinicians should interpret orthodontic abstracts with caution, particularly those addressing TMD-related interventions. Given clinicians’ demonstrated reliance on abstracts for rapid evidence appraisal, awareness of potential spin is critical to avoid misjudging treatment efficacy. Future meta-research should extend this comparative framework to other dental specialties to determine whether similar reporting gaps exist. Automated text-mining or natural-language-processing approaches could help detect spin more objectively and on a larger scale. Continuous monitoring of transparency metrics and abstract accuracy will be essential to sustaining research integrity and supporting evidence-based orthodontic practice. Enhancing reporting fidelity through preregistration, adherence to standardized frameworks, and transparent abstract presentation will not only improve reproducibility but also strengthen the clinical translation of orthodontic evidence into patient-centered outcomes.

## 5. Conclusions

Compared with general orthodontic research, TMD/TMJ-focused trials exhibited lower overall transparency, primarily due to limited protocol registration and inconsistent reporting-guideline adherence. Although the composite prevalence of abstract spin did not differ significantly between groups, TMD/TMJ studies were more likely to emphasize secondary benefits or recommend interventions despite non-significant results. These findings highlight persistent gaps in reporting integrity and interpretive neutrality within orthodontic research, particularly in TMD-related trials. Continued emphasis on trial registration, standardized reporting frameworks, and editorial oversight is essential to strengthen the reliability, transparency, and clinical applicability of the orthodontic evidence base.

## Figures and Tables

**Table 1:** Item-level reporting of transparency and spin by group, with odds ratios and 95% CIs.

Indicator	General % Yes	TMD/TMJ-Focused % Yes	OR (TMD/TMJ-Focused vs. General)	95% CI	*p*-Value
**Transparency items**					
Funding source noted	50.60	70.60	2.18	0.64–7.46	0.215
Risk of bias discussed	69.00	79.40	1.56	0.56–4.33	0.398
Confounders considered	83.60	73.50	0.49	0.20–1.16	0.106
Registered protocol/trial	55.50	26.50	0.15	0.07–0.30	<0.001
Followed reporting guidelines	71.40	58.80	0.38	0.21–0.69	0.001
**Spin items**					
Spin 1 (significant results only)	65.50	0.00	0.00	NA	<0.001
Spin 2 (claim efficacy despite NS)	7.90	8.8	1.14	0.34–3.78	0.829
Spin 3 (acknowledge NS but emphasize benefit)	37.00	73.5	4.62	1.90–11.22	<0.001
Spin 4 (recommend despite NS)	31.00	55.9	2.83	1.68–4.78	<0.001
Spin 5 (“trend toward significance”)	5.40	0.00	0.00	NA	<0.001

Note: Values represent the percentage of trials within each group in which the indicator was present. Odds ratios (ORs) compare TMD/TMJ-focused trials with general orthodontic trials and were estimated using logistic regression with cluster-robust standard errors by journal, adjusted for publication era and journal impact factor. ORs < 1 indicate that the indicator was less frequent in TMD/TMJ trials; ORs > 1 indicate that it was more frequent in TMD/TMJ trials. “NA” for the 95% confidence interval denotes complete separation (no TMD/TMJ trials with the given spin category), for which standard logistic models cannot provide a stable CI; in these cases, results should be interpreted descriptively. NS = non-significant. Spin categories correspond to: Spin 1, selective emphasis on statistically significant results; Spin 2, claims of efficacy despite non-significant results; Spin 3, acknowledgement of non-significant primary outcomes with emphasis on secondary benefits; Spin 4, recommendation of an intervention despite non-significant results; and Spin 5, “trend toward significance” language (see Section 2.5 for full operational definitions).

**Table 2:** Composite transparency and spin models from β-binomial mixed-effects analyses.

Outcome	Group OR (TMD/TMJ- Focused vs. General)	95% CI	*p*-Value	Predicted Proportion General	Predicted Proportion TMD/TMJ-Focused
Transparency (total)	0.58	0.34–0.99	0.047	0.821	0.729
Spin (total)	1.05	0.68–1.62	0.826	0.305	0.315

Note: Results from β-binomial mixed-effects models adjusted for publication era, natural-spline log-transformed journal impact factor, and random intercepts for journal. Predicted proportions represent adjusted mean transparency or spin scores on a 0–1 scale (equivalent to proportions of five indicators). ORs < 1 favor general orthodontic studies; ORs > 1 favor TMD/TMJ studies.

## Data Availability

The dataset supporting the findings of this study, including all study-level variables and coding decisions, is provided in Supplementary Table S3. Further details are available from the corresponding author upon reasonable request.

## Supplementary Materials

The following supporting information can be downloaded at: https://www.mdpi.com/article/10.3390/jcm14248907/s1, Table S1: Summary of full text screening omissions for general orthodontic literature; Table S2: Summary of full text screening omissions for TMD/TMJ-focused orthodontic literature; Table S3: Meta-analysis dataset. Table S4: Protocol amendments. Table S5: Summary of all included studies. Supplementary File S1: Database-Specific Search Strategies and Retrieval Counts. Supplementary Checklist: PRISMA 2020 Checklist.

## Abbreviations

The following abbreviations are used in this manuscript:

CI	Confidence interval
ICC	Intraclass correlation coefficients
OR	Odds ratio
RCT	Randomized controlled trial
TMD	Temporomandibular disorder
TMJ	Temporomandibular joint
