# Supplementary material for: Bias, Study Quality, and Confounding in Temporomandibular Disorder Research Compared to General Orthodontic Studies: A Systematic Review and Meta-Analysis
Source: J Clin Med. 2025 Dec 17;14(24):8907. doi: 10.3390/jcm14248907 (PMC12734166; doi:10.3390/jcm14248907)
Supplement: Supplementary file 1 [file jcm-14-08907-s001.zip › Supplementary File S1.pdf]

Supplementary File S1. Database-Specific Search Strategies and Retrieval Counts

Final search date: 15 October 2025. Counts reflect records retrieved at the final search (prior to deduplication).

A. General orthodontic trials (non-airway)

Database	Search strategy (verbatim)	Records retrieved (n)
PubMed/MEDLINE	((orthodontic[Title/Abstract] OR orthodontics[MeSH Terms] OR "orthodontic appliance"[Title/Abstract] OR "orthodontic appliances"[Title/Abstract] OR "interceptive orthodontics"[Title/Abstract] OR "clinical orthodontics"[Title/Abstract] OR "dental braces"[Title/Abstract] OR "dental bracket"[Title/Abstract])AND (randomized controlled trial[Publication Type] OR controlled clinical trial[Publication Type] OR randomized[Title/Abstract] OR randomised[Title/Abstract] OR trial[Title/Abstract] OR "clinical trial"[Title/Abstract] OR "split-mouth"[Title/Abstract] OR prospective[Title/Abstract] OR "quasi-randomized"[Title/Abstract] OR "quasi randomised"[Title/Abstract])) NOT (airway[Title/Abstract] OR "sleep apnea"[Title/Abstract] OR "upper airway"[Title/Abstract] OR OSA[Title/Abstract] OR snoring[Title/Abstract] OR breathing[Title/Abstract] OR respiratory[Title/Abstract] OR "pharyngeal airway"[Title/Abstract]) NOT (animals[MeSH Terms] NOT humans[MeSH Terms])	3040
WHO ICTRP	orthodontic OR orthodontics OR "orthodontic appliance" OR "interceptive orthodontics" OR "clinical orthodontics" OR "dental braces" OR "dental bracket"	1452

Embase	('orthodontics'/exp OR orthodontic*:ti,ab OR 'orthodontic appliance'/exp OR 'orthodontic appliance*':ti,ab OR 'interceptive orthodontics':ti,ab OR 'clinical orthodontics':ti,ab OR 'dental braces':ti,ab OR 'dental bracket*':ti,ab) AND ('randomized controlled trial'/exp OR 'controlled clinical trial'/exp OR randomized:ti,ab OR randomised:ti,ab OR trial:ti,ab OR 'clinical trial':ti,ab OR 'split-mouth':ti,ab OR prospective:ti,ab OR 'quasi-randomized':ti,ab OR 'quasi randomised':ti,ab) NOT (airway:ti,ab OR 'sleep apnea':ti,ab OR 'upper airway':ti,ab OR OSA:ti,ab OR snoring:ti,ab OR breathing:ti,ab OR respiratory:ti,ab OR 'pharyngeal airway':ti,ab) NOT ('animal'/exp NOT 'human'/exp)	1262
ClinicalTrials.gov	orthodontic OR orthodontics OR "orthodontic appliance" OR "interceptive orthodontics" OR "clinical orthodontics" OR "dental braces" OR "dental bracket"	817
CINAHL	(MH "Orthodontics+" OR orthodontic* OR "orthodontic appliance*" OR "interceptive orthodontics" OR "clinical orthodontics" OR "dental braces" OR "dental bracket*") AND (randomized OR randomised OR "controlled trial" OR "clinical trial" OR split-mouth OR prospective OR "quasi-randomized" OR "quasi randomised")	62

Total records retrieved across all sources: 6633

B. Temporomandibular disorder / temporomandibular joint (TMD/TMJ) orthodontic trials

Database	Search strategy (verbatim)	Records retrieved (n)
PubMed/MEDLINE	("Temporomandibular Joint Disorders"[Mesh] OR "Temporomandibular Joint"[Mesh] OR "Craniomandibular Disorders"[Mesh] OR	446

	temporomandibular[tiab] OR TMJ[tiab] OR TMD[tiab] OR craniomandibular[tiab]) AND ("Orthodontics"[Mesh] OR "Malocclusion"[Mesh] OR "Dental Occlusion"[Mesh] OR orthodontic[tiab] OR orthodontics[tiab] OR malocclusion[tiab] OR dentofacial[tiab] OR occlusion[tiab]) AND (therapy[tiab] OR treatment[tiab] OR intervention[tiab] OR appliance[tiab] OR splint[tiab] OR expansion[tiab] OR functional[tiab] OR mandibular[tiab] OR maxillary[tiab]) NOT ("Systematic Review"[Publication Type] OR "Meta-Analysis"[Publication Type] OR review[pt] OR editorial[pt] OR comment[pt] OR letter[pt] OR case reports[pt] OR protocol[tiab] OR arthroplasty[tiab] OR prosthesis[tiab] OR replacement[tiab]) NOT (animals[mh] NOT humans[mh])	
WHO ICTRP	(temporomandibular OR TMJ OR TMD OR craniomandibular) AND (orthodontic OR orthodontics OR malocclusion OR dentofacial OR occlusion) AND (therapy OR treatment OR intervention OR appliance OR splint OR functional OR expansion OR mandibular OR maxillary) NOT (arthroplasty OR prosthesis OR replacement)	179
Embase	('temporomandibular joint disorder'/exp OR 'temporomandibular joint'/exp OR temporomandibular:ti,ab OR TMJ:ti,ab OR TMD:ti,ab OR craniomandibular:ti,ab) AND ('orthodontics'/exp OR 'malocclusion'/exp OR 'dental occlusion'/exp OR orthodontic*:ti,ab OR malocclusion*:ti,ab OR dentofacial:ti,ab OR occlusion:ti,ab) AND (therapy:ti,ab OR treatment:ti,ab OR intervention:ti,ab OR appliance:ti,ab OR splint:ti,ab OR expansion:ti,ab OR functional:ti,ab OR mandibular:ti,ab OR maxillary:ti,ab) NOT	158

	('systematic review'/exp OR 'meta analysis'/exp OR review:ti,ab OR editorial:ti,ab OR comment:ti,ab OR letter:ti,ab OR 'case report'/exp OR protocol:ti,ab OR arthroplasty:ti,ab OR prosthesis:ti,ab OR replacement:ti,ab) AND [humans]/lim AND [clinical trial]/lim	
ClinicalTrials.gov	(temporomandibular OR TMJ OR TMD OR craniomandibular) AND (orthodontic OR orthodontics OR malocclusion OR occlusion OR dentofacial) AND (appliance OR splint OR functional OR mandibular OR maxillary OR expansion OR intervention OR treatment) NOT (arthroplasty OR prosthesis OR replacement)	127
CINAHL	(MH "Temporomandibular Joint Disorders+" OR MH "Temporomandibular Joint+" OR MH "Craniomandibular Disorders+" OR temporomandibular OR TMJ OR TMD OR craniomandibular) AND (MH "Orthodontics+" OR MH "Malocclusion+" OR MH "Dental Occlusion+" OR orthodontic OR orthodontics OR malocclusion OR dentofacial OR occlusion) AND (therapy OR treatment OR intervention OR appliance OR splint OR expansion OR functional OR mandibular OR maxillary) NOT (review OR "systematic review" OR "meta-analysis" OR editorial OR comment OR letter OR "case report" OR arthroplasty OR prosthesis OR replacement)	3

Total records retrieved across all sources: 913
